# Antibacterial Property and Biocompatibility of Silver, Copper, and Zinc in Titanium Dioxide Layers Incorporated by One-Step Micro-Arc Oxidation: A Review

**DOI:** 10.3390/antibiotics9100716

**Published:** 2020-10-20

**Authors:** Masaya Shimabukuro

**Affiliations:** Department of Biomaterials, Faculty of Dental Science, Kyushu University, 3-1-1 Maidashi, Higashi-ku, Fukuoka 812-8582, Japan; shimabukuro@dent.kyushu-u.ac.jp; Tel.: +81-92-642-6346

**Keywords:** titanium, biofilm, infection, micro-arc oxidation, silver, copper, zinc, antibacterial properties, coating, implant

## Abstract

Titanium (Ti) and its alloys are commonly used in medical devices. However, biomaterial-associated infections such as peri-implantitis and prosthetic joint infections are devastating and threatening complications for patients, dentists, and orthopedists and are easily developed on titanium surfaces. Therefore, this review focuses on the formation of biofilms on implant surfaces, which is the main cause of infections, and one-step micro-arc oxidation (MAO) as a coating technology that can be expected to prevent infections due to the implant. Many researchers have provided sufficient data to prove the efficacy of MAO for preventing the initial stages of biofilm formation on implant surfaces. Silver (Ag), copper (Cu), and zinc (Zn) are well used and are incorporated into the Ti surface by MAO. In this review, the antibacterial properties, cytotoxicity, and durability of these elements on the Ti surface incorporated by one-step MAO will be summarized. This review is aimed at enhancing the importance of the quantitative control of Ag, Cu, and Zn for their use in implant surfaces and the significance of the biodegradation behavior of these elements for the development of antibacterial properties.

## 1. Current Clinical Issues Related to Titanium (Ti)

Titanium (Ti) and its alloys are used for devices requiring strength, elongation, and long-term bone bonding in orthopedics, cardiovascular medicine, dentistry, and other applications owing to their high corrosion resistance, specific strength, and tissue compatibility [1]. Compared with other metals, Ti has the unique property of osseointegration. Osseointegration is defined as “the formation of a direct interface between an implant and bone, without intervening soft tissue. No scar tissue, cartilage, or ligament fibers are present between the bone and implant surface. The direct contact of bone with implant surface can be verified microscopically” [2]. The hard-tissue compatibility of Ti is caused by osseointegration. In other words, the reaction that occurs at the interface between Ti and bone tissue promotes bone formation and bone binding. These are the well-known advantages of using Ti in implant materials compared with other metals. The interface between Ti and bone tissue is apparent soon after implantation at the micrometer and nanometer scales [3,4,5,6,7,8]. The Ti surface is covered by an amorphous titanium oxide (TiO_2_) layer and shows a high corrosion resistance in vivo [9,10,11,12,13] along with a low toxicity. Therefore, the Ti surface is crucial as a beneficial interface between Ti and the bone tissue.

However, even with a high hard-tissue compatibility, several clinical issues have been reported for Ti and are summarized in Table 1. The disadvantages of metals for their use as biomaterials include their artificial nature and lack of bio-functional properties. The exploration of novel and advanced strategies to prevent these clinical issues is an active area of research. Among these, biomaterial-associated infections on Ti implants have been recognized as devastating and threatening complications for patients and orthopedists, and many researchers have focused on this issue. This review focusses on one-step micro-arc oxidation (MAO), which is one of the coating techniques that is expected to prevent biomaterial-associated infections.

## 2. Biomaterial-Associated Infections

Biomaterials are used in implants and devices in medical applications that are used in diagnosis and temporary support or the permanent restoration of human function. These applications have always been hindered by the development of biomaterial-associated infections linked to implant/device use [14,15,16,17,18]. The current incidence rates of infections in various implant/device applications are well summarized in [19]. Infections can occur on all biomaterial surfaces. The onset of biomaterial-associated infections is frequently accompanied by patient morbidity and discomfort, and can lead to the surgical replacement of the implant after lengthy and unsuccessful attempts to mitigate infections using antibiotics. This clinical routine incurs additional health care costs and patient morbidities. Revision surgery to replace a total hip arthroplasty triples the cost of the primary implant procedure and amounts to an average of US $75,000 [20,21].

**Figure 1:**
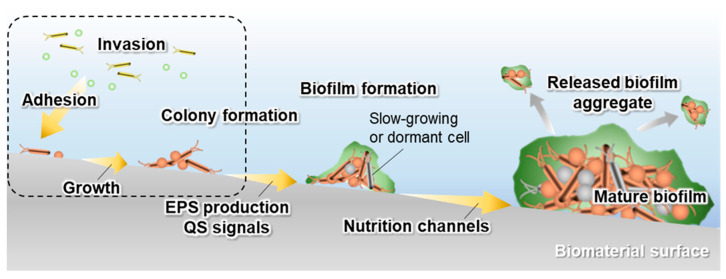
Schematic diagram of the biofilm formation process. The dashed area represents the initial stages of biofilm formation.

It is essential to prevent the formation of biofilms, which are the main cause of biomaterial-associated infections. Biofilms are hallmarks of extracellular polymeric matrix production [22]. Many bacteria that form biofilms produce a variety of extracellular polymeric substances (EPSs). The establishment of a biofilm is the final state of bacterial infections that can persist despite treatment. Biofilms are more resistant to a variety of antimicrobial agents, including antibiotics, owing to the three-dimensional structure of the biofilm and the physiology of the adherent bacteria [23,24,25,26,27]. The mechanism of biofilm formation on biomaterial surfaces is shown in Figure 1.

Biofilms are generally formed as a result of bacterial adhesion, growth, colony formation, EPS production, quorum sensing (QS) signals, and the formation of nutrition channels. Bacterial invasion can occur due to implant surgery or the hematogenous spread of bacteria. The invading bacteria initially adhere to the biomaterial surface through cell surface-associated adhesions [27,28]. The attached bacteria can proliferate and form colonies. During this time, an EPS matrix is generated. The chemical and physical composition of the EPS matrix varies between species and growth conditions [27]. Regardless of these differences, the outcome of EPS production typically is the enhanced adhesion of the bacteria embedded in the matrix. In addition, the interaction between bacteria and eukaryotic cells is promoted—for example, the matrix components produced by some bacteria are required for the adhesion of bacteria to a variety of protein components of the host cells at the onset of infection [29,30]. In addition, the EPS matrix protects bacteria from antibiotics, antimicrobial agents, and host innate immune components [23,31,32,33]. Thus, the EPS matrix is a multi-functional and protective scaffold that forms specific chemical and physical microenvironments. Within the biofilm, bacteria develop a quorum-sensing system for communication. Quorum sensing is the regulated gene expression that occurs in response to fluctuations in cell density [34]. The quorum sensing process controls and optimizes a variety of activities and leads to bacterial diversity in biofilms. The result is often the recalcitrance of implant-related biofilm infections to antibiotic treatments. Mature biofilms can release aggregates of EPSs that can spread the infection. Often, the only way to eradicate the infection and prevent sepsis is to remove the contaminated device from the patient. To avoid this, biofilm formation must be prevented by inhibiting the initial stage of biofilm formation during the device implantation. Therefore, the implant materials require antibacterial properties to inhibit this stage.

## 3. Strategies to Prevent Biofilm Formation by Coatings

Novel and effective strategies for the prevention of biofilm formation on biomaterials have become an active area of research. Surface functionalization based on surface coating technology aims to inhibit the initial stages of biofilm formation—namely, bacterial invasion, adhesion, and growth; in particular, anti-biofouling (or anti-adhesive) properties and antibacterial properties are key bio-functional properties. Anti-biofouling, which relies on the chemical and physical properties of polymers, can control biofilm formation in the absence of antibacterial agents [35,36,37,38,39,40].

The antibacterial strategy has become a very active area of research for the prevention of biofilm formation. Antibacterial agents can be incorporated on the device surface. Bacteria that encounter the surface can be killed, nullifying their adhesion and growth. Antibiotics, peptides, enzymes, organic cation compounds, non-organic compounds, and elements have been used as antibacterial agents. Among them, silver (Ag), copper (Cu), and zinc (Zn) have been extensively used as major antibacterial elements because of their excellent antibacterial effects. Since the antibacterial properties and host cell toxicities of these elements are dose-dependent, it is important to control the concentration of antibacterial elements to kill the bacteria on implant surfaces with minimal or no harmful effects on the living host tissue [41,42,43,44,45,46,47,48,49,50,51,52,53]. Therefore, coating technologies play an important role in the fabrication of antibacterial surfaces because they can easily affect the surface composition of implants.

## 4. Micro-Arc Oxidation (MAO)

**Figure 2:**
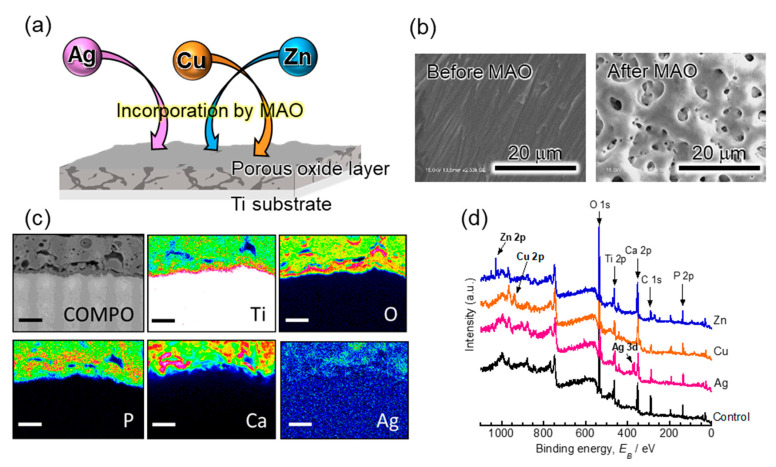
(**a**) Schematic diagram of the formation of a porous oxide layer on the Ti substrate and the incorporation of antibacterial elements by micro-arc oxidation (MAO). (**b**) Scanning electron microscopy (SEM) images of the Ti surface before and after MAO; (**c**) cross-sectional views of Ag-incorporated porous oxide layer and element mapping results by electron probe micro analyzer (EPMA). Scale bar represents 10 µm; (**d**) X-ray photoelectron spectroscopy (XPS) survey scan spectra obtained from the specimen, with and without antibacterial elements. The spectra obtained from the control (specimen without antibacterial elements), Ag-, Cu-, and Zn-incorporated specimens are shown from the bottom to the top. Specimens were MAO-treated at 400 V using the electrolytes containing 150 mM of calcium acetate and 100 mM of calcium glycerophosphate with or without 2.5 mM of silver nitrate, 2.5 mM of copper chloride, or 2.5 mM of zinc chloride.

MAO, which is also termed anodic spark deposition (ASD) or plasma electrolytic oxidation (PEO), is an electrochemical treatment for valve metals. MAO is a wet process that is performed at high voltages using a specific electrolyte. A hard and thick porous oxide layer forms on the metallic substrate, and MAO allows the elements comprising the electrolyte to be incorporated into the oxide layer. This layer has a high adhesive strength compared to various coating layers because the oxide layer grows in the direction of the substrate. Many studies have reported the advantages of MAO for Ti-based materials for medical devices. MAO improves the hard-tissue compatibility of Ti when the electrolyte contains calcium (Ca) and phosphate (P). The biocompatibility of MAO coatings has been demonstrated by numerous in vitro and in vivo tests [54,55,56,57,58,59,60,61,62,63,64,65,66,67]. On the other hand, since 2009 MAO has been used to incorporate antibacterial elements onto the Ti surface to generate an antibacterial surface. A schematic diagram of the incorporation of Ag, Cu, and Zn by MAO and their surface characterization results are shown in Figure 2. MAO easily and selectively incorporates the required elements into the titanium surface owing to the compositional control in the electrolyte. Table 2 summarizes the published articles related to the one-step MAO-based incorporation of antibacterial elements onto the Ti surface [68,69,70,71,72,73,74,75,76,77,78,79,80,81,82,83,84,85,86,87,88,89,90,91,92,93,94,95,96,97,98,99,100,101,102]. It should be noted that some researchers have combined MAO and other surface modification processes [103,104,105,106]. Many researchers have used one-step MAO to incorporate antibacterial elements onto Ti surfaces and have provided sufficient data to prove the efficacy of this approach. The MAO treatment of Ti using electrolytes containing antibacterial elements has proven effective against various bacteria, including *Escherichia coli* (*E. coli*), *Staphylococcus aureus* (*S. aureus*), methicillin-resistant *Staphylococcus aureus* (MRSA), *Actinobacillus actinomycetemcomitans* (*A. actinomycetemcomitans*), *Streptococcus mutans* (*S. mutans*), and *Pseudomonas aeruginosa* (*P. aeruginosa*). In addition, the structure of the titanium dioxide layer formed by MAO captures the bacteria and leads to the trap-killing system on the implant surface [106]. Therefore, the typical porous structure formed by MAO is beneficial for the development of antibacterial properties.

## 5. Dual-Functionalization by MAO

**Figure 3:**
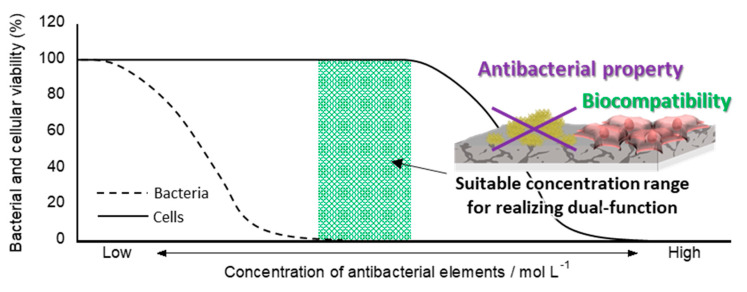
Conceptual diagram of the suitable concentration range (green-colored area) of antibacterial elements for the dual-functionalization of the Ti surface.

Ag, Cu, and Zn in the oxide layer develop both antibacterial properties and cytotoxicity in a dose-dependent manner. Ning et al. [107] reported that suitable concentration ranges of Ag, Cu, and Zn ions can kill both *S. aureus* and *E. coli* without harmful effects on fibroblasts. In the case of implant surfaces, antibacterial properties and absence of harmful effects on osteoblasts play an important role in preventing infection and bone reconstruction. Therefore, it is necessary to realize the importance of both antibacterial properties and biocompatibility on the Ti surface—namely, dual-functionalization by MAO. A conceptual diagram of the dual-functionalization of the Ti surface is shown in Figure 3. The half maximal inhibitory concentrations (IC_50_s) of Ag, Cu, and Zn ions against MC3T3-E1 cells were 2.77, 15.9, 90.0 µM, respectively [53]. Therefore, Ag should be the most toxic element in osteoblasts. However, previous studies have revealed that a slight amount of Ag did not affect the osteoblast activity [68,78,82,96,100]. Moreover, the specimen with suitable amounts of Ag had no influence on differentiation and accelerated the calcification [96]. Therefore, the incorporation of a suitable amount of Ag by MAO achieved a dual function on the Ti surface. The biocompatibility of Cu-incorporated specimens was evaluated using a wide variety of cells. The large amount of Cu developed cytotoxicity in osteoblast [81] and fibroblast cells [77], and the specimen with a suitable amount of Cu exhibited a good interaction with hard and soft tissues. A slight amount of Cu on the Ti surface promoted the responses of osteoblasts, fibroblasts, and endothelial cells [70,77,81,86,90,99,101]. Huang et al. reported that the Cu-incorporated surface contributed to the improvement of both the macrophage-mediated bone formation and bactericidal capacity [86]. In addition to the incorporation of other elements, large amount sof Zn on the Ti surface exhibited cytotoxicity against osteoblasts, and a suitable amount of Zn promoted its response [71,85,95,97]. Hence, the quantitative control of the incorporation of Ag, Cu, and Zn plays a key role in the dual-functionalization of the Ti surface by MAO. In addition, some researchers have reported that the surface structure is one of the key factors in the osteoinductive ability [108]. Therefore, the typical porous structure formed by MAO may be influenced for the improvement of the osteoinduction of implants.

## 6. Time-Transient Effects of Ag, Cu, and Zn on Their Antibacterial Properties

**Figure 4:**
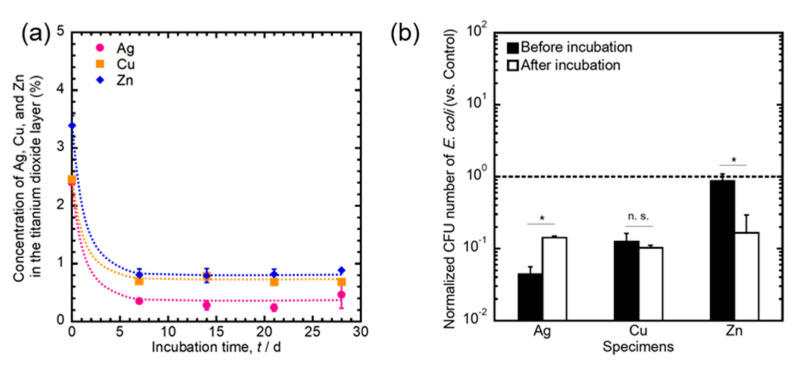
Changes in the concentration of Ag, Cu, and Zn in the oxide layer (**a**) and antibacterial effects (**b**) before and after incubation in saline for 28 days [97,102].

Prosthetic joint infections are generally categorized into two types: early infections (within 3 months after surgery) and late infections (12 months after surgery). The main cause of late infections is bacterial invasion due to hematogenous spread from another site [109]. On the other hand, Coventry et al. sorted late infection into three types: early postoperative infection (infection occurring within less than 1 month after insertion), late acute infection (infection occurring more than 1 month after insertion, with symptom duration less than 7 days), or late insidious infection (infection occurring more than 1 month after insertion, with symptom duration more than 7 days) [110]. Therefore, the antibacterial surface should be designed to prevent long-term infections. In particular, the long-term inhibition of biofilm formation relies on the durability of antibacterial effects. Surface changes are key in the development of antibacterial effects. Therefore, the biodegradation behavior must be precisely characterized to understand its antibacterial effect and durability. Our previous studies [97,102] simulated the biodegradation behaviors of Ag-, Cu-, and Zn-incorporated Ti surfaces by immersion in physiological saline for 28 days to investigate the changes in surface composition, chemical states, and antibacterial effects. The surface concentrations of Ag, Cu, and Zn were dramatically decreased by incubation for up to 7 days and remained at slight amounts until 28 days (Figure 4a). The antibacterial effect of Ag-incorporated specimens was weakened, the effect of Cu was maintained, and the effect of Zn was improved after 28 days of incubation in saline (Figure 4b). The antibacterial effects of these elements relied on their changes in chemical states. After incubation in saline, the chemical states of Ag, Cu, and Zn in the oxide layers changed as follows: Ag_2_O to Ag, Cu_2_O (stable), and Zn^2+^ to ZnO (Table 3). Hence, the biodegradation behavior of antibacterial surfaces must be considered for both understanding their durability and preventing late infections.

## 7. Conclusions

Biomaterial-associated infections are caused by the formation of biofilms on implant surfaces and are still an important clinical issue. The incorporation of antibacterial elements by MAO is an effective approach for preventing biofilm formation owing to the dual-functionalization of the Ti surface based on the quantitative control of the incorporated elements.

The incorporation of Ag by MAO leads to both the highest antibacterial effect and the highest cytotoxic risk. However, the quantitative control of the incorporation of Ag leads to the realizing both antibacterial property and biocompatibility. The biodegradation behavior of Ag incubated in saline could affect the durability of the antibacterial effects.

The antibacterial effects and the cytotoxic risks caused by the incorporation of Cu and Zn are lower than those of Ag. Moreover, suitable amounts of these elements promote osteoblast responses. The antibacterial effect of Cu remains for at least 28 days, and that of Zn was developed after 28 days of incubation in saline. These specific phenomena will help the control of antibacterial effects on the implant surface in the long term.

However, the durability of the antibacterial effect remains unclear and is necessary for preventing late infections. Based on the prosthetic joint infections classification, the antibacterial effect should remain for at least 3 months, and ideally for 1 year. The antibacterial surface on implants should be controlled and designed based on the biodegradation behaviors in the body. Finally, the author hopes that the incorporation of antibacterial elements by MAO can help to eradicate biomaterial-associated infections.

## Figures and Tables

**Table 1:** Current clinical issues related to Ti use in the body.

Problem	Medical Devices
Stress shielding	Bone plate; stem of artificial joint
Adhesion to bone	Bone screw; bone nail
Cracking and fracture by excessive deformation	Spinal rod; maxillofacial plate
Crevice corrosion; pitting	Stent graft
Fracture	Endodontic file
Corrosion with fluoride	Dental restorative
Cytotoxicity	All devices
Biomaterial-associated infections; peri-implantitis; prosthetic joint infection	Abutment of dental implant; orthodontic implant anchor; percutaneous device; screw of external bone fixator; artificial joint

**Table 2:** Summary of the published articles related to the incorporation of antibacterial elements onto the Ti surface by one-step MAO.

Year	Authors	Elements	Tested Bacteria	Tested Cells
2009	Song et al. [68]	Ag and Pt	*S. aureus* and *E. coli*	HOS and MG63
2013	Lin et al. [69]	Bi	*S. aureus*, MRSA, and *A. actinomycetemcomitans*	MG63
	Zhu et al. [70]	Cu	*S. aureus*	MG63
	Zhao et al. [71]	Zn	*S. mutans*	MG63
2014	Zhao et al. [72]	Zn	-	-
	Yu et al. [73]	Mn	*S. aureus* and *E. coli*	rBMMSC
	Yao et al. [74]	Cu	*S. aureus* and *E. coli*	-
2015	Teker et al. [75]	Ag	*S. aureus* and *E. coli*	-
2016	Rokosz et al. [76]	Cu	-	-
	Zhang et al. [77]	Cu	*S. aureus*	L-929
	He et al. [78]	Ag	*S. aureus* and *E. coli*	MC3T3-E1
	Zhang et al. [79]	Zn and Ag	*S. aureus*	-
2017	Rokosz et al. [80]	Cu	-	-
2018	Zhang et al. [81]	Cu	*S. aureus*	MC3T3-E1 and Endothelial cells
	Aydogan et al. [82]	Ag	*S. aureus*	Saos-2
	Zhang et al. [83]	Cu and Zn	*S. aureus*	L-929
	Roknian et al. [84]	Zn	*S. aureus* and *E. coli*	-
	Sopchenski et al. [85]	Zn	*S. aureus* and *P. aeruginosa*	ADSCs
	Huang et al. [86]	Cu	*S. aureus*	RAW 264.7 and Saos-2
	Sopchenski et al. [87]	B	*S. aureus* and *P. aeruginosa*	ADSCs
	Zhou et al. [88]	F	*S. aureus* and *E. coli*	Rabbit MSC
	Du et al. [89]	Zn	*S. aureus* and *E. coli*	-
2019	Zhao et al. [90]	Cu and F	*S. aureus*	MC3T3-E1
	Li et al. [91]	Fe	*S. aureus*	L929
	Zhang et al. [92]	Cu	-	-
	Zhou et al. [93]	Co and F	*S. aureus* and *E. coli*	MSCs
	Zhang et al. [94]	Zn	*E. coli*	-
	Zhang et al. [95]	Zn	*E. coli*	MC3T3-E1
	Shimabukuro et al. [96]	Ag	*S. aureus* and *E. coli*	MC3T3-E1
	Shimabukuro et al. [97]	Zn	*E. coli*	MC3T3-E1
2020	Zhang et al. [98]	Ag	*E. coli*	-
	Zhang et al. [99]	Cu	*S. aureus*	MC3T3-E1
	Zhang et al. [100]	Ag	*S. aureus*	MC3T3-E1
	Shimabukuro et al. [101]	Cu	*S. aureus* and *E. coli*	MC3T3-E1
	Shimabukuro et al. [102]	Ag and Cu	*E. coli*	-

**Table 3:** Summary of the binding energy (*E_B_*), kinetic energy (*E_K_*), and modified Auger parameter (α’) values obtained from Ag-, Cu-, and Zn-incorporated oxide layers before and after incubation in saline for 28 days [97,102].

Elements	Incubation Time/d	*E_B_*/eV	*E_K_*/eV	α’/eV	Chemical State
Ag	0	368.1	356.8	724.9	Ag_2_O
	28	368.5	357.6	726.1	Ag
Cu	0	933.1	916.5	1849.6	Cu_2_O
	28	933.3	916.2	1849.5	Cu_2_O
Zn	0	1025.8	983.6	2009.4	Zn^2+^
	28	1024.9	985.4	2010.3	ZnO
